# Brain metabolic signatures in patients with genetic and nongenetic amyotrophic lateral sclerosis

**DOI:** 10.1111/cns.14193

**Published:** 2023-03-27

**Authors:** Pan Liu, Yongxiang Tang, Wanzhen Li, Zhen Liu, Ming Zhou, Jian Li, Yanchun Yuan, Liangjuan Fang, Jifeng Guo, Lu Shen, Hong Jiang, Beisha Tang, Shuo Hu, Junling Wang

**Affiliations:** ^1^ Department of Neurology, Xiangya Hospital Central South University Changsha China; ^2^ Department of Neurology The Central Hospital of Shaoyang Shaoyang China; ^3^ Department of Nuclear Medicine, Xiangya Hospital Central South University Changsha China; ^4^ National Clinical Research Center for Geriatric Diseases, Xiangya Hospital Central South University Changsha China; ^5^ Key Laboratory of Hunan Province in Neurodegenerative Disorders Central South University Changsha China; ^6^ Center for Medical Genetics, School of Life Sciences Central South University Changsha China; ^7^ Engineering Research Center of Hunan Province in Cognitive Impairment Disorders Central South University Changsha China; ^8^ Hunan International Scientific and Technological Cooperation Base of Neurodegenerative and Neurogenetic Diseases Changsha China; ^9^ Key Laboratory of Biological Nanotechnology of National Health Commission, Xiangya Hospital Central South University Changsha China

**Keywords:** ^18^F‐FDG‐PET, amyotrophic lateral sclerosis, brain metabolism, genetic, whole exome sequencing

## Abstract

**Aims:**

To study the brain metabolic signature in Chinese amyotrophic lateral sclerosis (ALS) patients and compare the difference in brain metabolic patterns between ALS with and without genetic variants.

**Methods:**

We included 146 patients with ALS and 128 healthy controls (HCs). All patients with ALS underwent genetic testing to screen for ALS related genetic variants and were then divided into genetic (*n* = 22) and nongenetic ALS (*n* = 93) subgroups. All participants underwent brain ^18^F‐FDG‐PET scans. Group comparisons were performed using the two‐sample *t*‐test model of SPM12.

**Results:**

We identified a large of hypometabolic clusters in ALS patients as compared with HCs, especially in the bilateral basal ganglia, midbrain, and cerebellum. Moreover, hypometabolism in the bilateral temporal lobe, precentral gyrus and hypermetabolism in the left anterior cingulate, occipital lobe, and bilateral frontal lobe were also found in ALS patients as compared with HCs. Compared with nongenetic ALS patients, genetic ALS patients showed hypometabolism in the right postcentral gyrus, precuneus, and middle occipital gyrus. The incidence of sensory disturbance in patients with genetic ALS was higher than that in patients with nongenetic ALS (5 of 22 [22.72%] vs. 7 of 93 [7.52%], *p* = 0.036).

**Conclusions:**

Our investigation provided unprecedented evidence of relative hypometabolism in the midbrain and cerebellum in ALS patients. Genetic ALS patients showed a specific signature of brain metabolism and a higher incidence of sensory disturbance, indicating that genetic factors may be an underlying cause affecting the brain metabolism and increasing the risk of sensory disturbance in ALS.

## Graphical abstract

This is the first study to measure compare the difference in brain metabolic patterns between ALS with and without genetic variants. Interestingly, we found that genetic ALS patients showed a specific signature of brain metabolism and a higher incidence of sensory disturbance, indicating that genetic factors may be an underlying cause affecting the brain metabolism and increasing the risk of sensory disturbance in ALS.

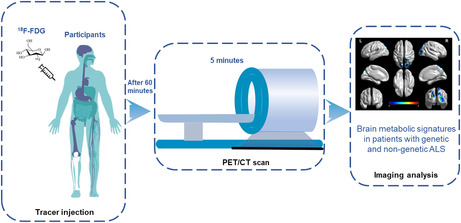

## 1 INTRODUCTION

Amyotrophic lateral sclerosis (ALS) is a rare neurodegenerative disorder characterized by weakness and atrophy due to loss of both upper and lower motor neurons, causing death due to respiratory paralysis within 3‐5 years from the onset.^1^ Although its etiology is still poorly understood, the interaction between genetic background, environmental, and lifestyle factors is a potential cause of ALS.^2^ About 10% of cases are familial ALS (fALS), while the remaining 90% of cases are sporadic ALS (sALS). Since the *SOD1* gene was reported in 1993, more than 40 genes have been reported to be linked with ALS.^1^, ^2^, ^3^, ^4^ ALS patients carrying specific mutations or variants may show distinct clinical phenotypes and prognosis.^5^ Uncovering the relationship between genotype and phenotype has important implications for pathogenetic explanations in ALS.

^18^F‐fluorodeoxyglucose positron emission tomography (^18^F‐FDG‐PET) is a powerful tool to display the brain metabolic signature in ALS.^6^, ^7^, ^8^, ^9^ Previous studies have shown that different ALS phenotypes displayed their specific brain metabolic changes. For example, ALS with cognitive impairment or frontotemporal dementia (FTD) demonstrated prefrontal, anterior cingulate, and insular hypometabolism when compared with ALS with normal cognition.^10^, ^11^, ^12^ However, a large ^18^F‐FDG‐PET study is still lacking in Chinese mainland. Moreover, most ^18^F‐FDG‐PET studies focused on the metabolic features in characterize patients carrying GGGGCC repeat expansion in *C9orf72*,^13^, ^14^, ^15^ While the GGGGCC repeat expansion is rarely found in ALS patients in Asia, especially in China.^16^ Moreover, genome‐wide pathogenic mutation metabolic pattern in ALS has not been systematically studied yet. Herein, the aim of this study was twofold: first, to elucidate the brain metabolic pattern in ALS patients in the mainland of China; second, to explore the brain metabolic changes characterizing genetic ALS as compared with nongenetic ALS.

## 2 MATERIALS AND METHODS

### 2.1 Participants

The present study recruited a cohort of 146 patients with ALS from the Department of Neurology, Xiangya Hospital, Central South University from January 1, 2014, to January 1, 2021. All patients with ALS were diagnosed with clinically definite‐, probable‐, or probable laboratory‐supported ALS by at least two experienced neurologists according to the revised El Escorial criteria 2015.^17^ Demographic and clinical data of ALS, including age at onset (AAO), age at PET, sex, family history, site of onset, disease duration, and ALS Functional Rating Scale‐Revised (ALSFRS‐R) scores were collected by specialists. ALS patients with sensory symptoms or abnormal sensory nerve conductions detected by electromyogram were regarded as ALS patients with sensory disturbances. We enrolled 128 sex‐ and age‐matched healthy controls (HCs) from the Department of Nuclear Medicine in Xiangya Hospital, of which, healthy was defined as (i) absence of no oncologic disease, (ii) with brain ^18^F‐FDG‐PET scan reported as normal by at least two nuclear medicine doctors, (iii) with normal neurological examination, and (iv) lack of a history of neurological diseases.

### 2.2 Genetic analysis

In this study, 115 patients with ALS in this study underwent genetic testing by whole exome sequencing (WES), standard polymerase chain reaction (PCR) and repeat primed polymerase chain reaction (RP‐PCR) assay. The genomic data was stored in our inhouse data base (National Geriatric Clinical Medical Research Center, Xiangya Hospital, Bioinformatics Center). According to previous studies (Table S1),^2^, ^18^ we focused on 52 known ALS related pathogenic genes, of which we used WES examined 50 genes. WES was completed by the Illumina HiSeq4000 using the Agilent SureSelect Human All Exon V6. Variants were annotated by software (UCSC hg19).^19^ The criteria used to define rare disease variants (RDVs) referred to the previous research.^18^ Variant frequencies were initially determined in 1000 Genomes, esp6500s, gnomAD, ExAC, and our inhouse WES data of 1258 controls without any nervous neurological disease to draft rare single nucleotide polymorphisms (SNP). Heterozygous variants in dominant ALS‐causative genes with a minor allele frequency (MAF) less than 0.1% across all population databases. Homozygous or compound heterozygous variants in recessive ALS‐causative genes with a MAF less than 1% across all population databases. All stop gain/loss, frameshift, and splice‐site variants (falling within 2 bp of exon‐intron junctions) were selected for further analysis. Rare missense variants are functionally predicted to be deleterious with a ReVe value more than 0.7.^20^ Polynucleotide repeat expansions in the *ATXN2* and *C9orf72* genes were detected by standard PCR and RP‐PCR assay. The CAG repeat expansion more than 27 in *ATXN2* was consider ALS susceptibility, and the GGGGCC repeat expansion more than 30 in *C9orf72* was considered pathological. We define genetic variant ALS group as ALS patients who carry RDVs in the known ALS genes, nongenetic variant ALS group as ALS patients who do not carry RDVs in the known ALS genes.

### 2.3 ^18^F‐FDG‐PET imaging acquisition

Brain ^18^F‐FDG‐PET was performed according to the published criteria.^21^ Participants fasted at least 6 h before the examination. The fasting blood glucose was lower than 7.2 mmoL/L in all subjects before the test. A dose of 3.7 MBq/kg of ^18^F‐FDG was injected intravenously through the cubital vein over 1 min. The PET images were acquired in three dimensions for 5 min, starting at 60 min after intravenous ^18^F‐FDG injection. The full width of the scan at half‐maximum was 5.4 mm. PET/computed tomography (CT) images were acquired by a Discovery Elite PET/CT scanner (GE Healthcare, Waukesha, USA). Participants were placed in the PET scanner so that slices were parallel to the canthomeatal line. All images were reconstructed as a 256 × 256 trans‐axial matrix using the 3D VUE Point (GE Healthcare, Waukesha, USA) ordered‐subset expectation–maximization algorithm with 6 iterations and 6 subsets, which produced 47 trans‐axial images at 3.25‐mm intervals. A low‐dose CT scan was obtained simultaneously for photon attenuation correction. Participants were also monitored on‐site for other signs of adverse effects for 90 min after injection of ^18^F‐FDG and asked to report any ensuing adverse effects.

### 2.4 Statistical analysis

Data are shown as the mean (standard deviation, SD) or percentage. Statistical analysis was performed using SPSS v25.0 (IBM, New York, USA). Differences with *p* < 0.05 were considered statistically significant. Kolmogorov–Smirnov tests were utilized to check the normality of the clinical data. All data should be subject to tests for normality. The demographic and clinical characteristics were compared as follows. The *χ* ^2^ test was used to analyze the differences among categorical variables. The differences among quantitative, continuous variables were analyzed with Student's *t*‐test or the Mann–Whitney *U* test.

Image processing was performed using the SPM12 toolkit (https://www.fil.ion.ucl.ac.uk/spm/) implemented in MATLAB R2013b (MathWorks). Individual ^18^F‐FDG‐PET image volumes were spatially normalized into standard stereotactic Montreal Neurological Institute (MNI) space, resliced to 2 × 2 × 2 mm. An 8‐mm full‐width half‐maximum Gaussian kernel was used to improve between‐participant spatial alignment and smooth data for statistical analysis.^22^ Once the images were spatially normalized and smoothed, a general linear model was used to carry out the appropriate voxel‐by‐voxel univariate statistical tests. Image intensity was normalized between participants to prevent interparticipant variability in cerebral tracer uptake from masking regional changes.^22^ This was done using proportional scaling, which scales each image proportionally to the mean global brain activity.

Comparisons between different groups were performed using the two‐sample *t*‐test model of SPM12. When each patient's group was compared with HCs, sex, age at PET, and years of education were used as covariates. In the comparison of subgroups of patients with ALS, sex, age at PET, disease duration, years of education, and ALSFRS‐R score were used as covariates. The height threshold of metabolic changes was set at *p* ˂ 0.001 (*p* ˂ 0.05 Family‐wise error [FEW]‐corrected at cluster). If no cluster of significant difference was identified, a height threshold of *p* < 0.005 (*p* < 0.05 FWE‐corrected at cluster) was set to perform further exploratory analyses. After data was preprocessed using SPM12, significant clusters were visualized, reported, and anatomically labeled using the xjView (http://www.alivelearn.net/xjview) and BrainNet Viewer.^23^

### 2.5 Ethics approval

Written informed consent was obtained from all participants, and the study protocol was approved by the Ethics Committee and the Expert Committee of Xiangya Hospital, Central South University.

## 3 RESULTS

### 3.1 Demographic and clinical features of subjects

The demographic and clinical features of all subjects are listed in Table 1, Table S5 and Table S6. In the ALS cohort, the mean AAO was 54.84 ± 10.22 years, the mean age at PET is 55.90 ± 10.10 years (97 male, 49 female). The mean disease duration was 14.44 ± 15.11 months. There were 34 ALS patients with bulbar onset and 112 ALS patients with spinal onset. The mean ALSFRS‐R score was 38.86 ± 6.60. Patients with ALS and HCs were well matched for age and sex as shown in Table 1. There was no significant difference between the genetic group and nongenetic group in terms of age at PET, AAO, sex, site of onset, disease duration, years of education, diagnostic categories, and ALSFRS‐R score (Table 1). The incidence of sensory disturbance in patients with genetic ALS was higher than that in patients with nongenetic ALS (*χ* ^2^ test, 5 of 22 [22.72%] vs. 7 of 93 [7.52%], *p* = 0.036).

**TABLE 1:** Demographic and clinical features of genetic ALS, nongenetic ALS, and HCs.

	Total ALS patients	HCs	*p*	Genetic ALS	Nongenetic ALS	*p*
Number	146	128		22	93	
Age at PET (years)	55.90 ± 10.10	55.24 ± 7.75	0.545^a^	52.18 ± 10.93	56.31 ± 9.23	0.072^a^
Sex						
Male (%)	97 (66.4)	88 (68.8)	0.684	18 (81.8)	60 (64.5)	0.118^c^
Female (%)	49 (33.6)	40 (31.2)		4 (18.2)	33 (35.5)	
Years of education	8.75 ± 3.45	8.82 ± 2.77	0.781^a^	8.12 ± 2.49	8.85 ± 2.62	0.314^a^
Age at onset (years)	54.84 ± 10.22			51.50 ± 10.97	55.29 ± 9.55	0.107^b^
Diagnostic category (Revised El Escorial criteria 2015)						
Definite ALS (%)	32 (21.9)			4 (18.1)	22 (23.7)	0.160^c^
Probable ALS (%)	31 (21.2)			2 (9.1)	23 (24.7)	
Laboratory support probable ALS (%)	83 (56.9)			16 (72.8)	48 (51.6)	
Site of onset						
Bulbar onset (%)	34 (23.29)			4 (18.2)	26 (28.0)	0.348^c^
Spinal onset (%)	112 (76.71)			18 (81.8)	67 (72.0)	
Disease duration (months)	14.44 ± 15.11			9.91 ± 12.50	14.01 ± 11.62	0.145^b^
ALSFRS‐R	38.86 ± 6.60			40.00 ± 5.07	38.72 ± 6.07	0.362^b^
Sensory symptoms (%)						
Positive	12 (8.2)			5 (22.7)	7 (7.5)	**0.036** ^c^
Negative	134 (97.8)			17 (77.3)	86 (92.5)	
Genetic status						
*SOD1*	5 (5/115)			5 (5/22)		
*OPTN*	3 (3/115)			3 (3/22)		
Other genes	14 (14/115)			14 (14/22)		

Abbreviations: ALS, Amyotrophic lateral sclerosis; ALSFRS‐R, Amyotrophic Lateral Sclerosis Functional Rating Scale‐Revised; HCs, healthy controls.

^a^ Student's *t*‐test, *p* < 0.05 was considered significant.

^b^ Mann–Whitney *U* test, *p* < 0.05 was considered significant.

^c^ *χ* ^2^ test, *p* < 0.05 was considered significant. Bold value indicate statistically significant differences with P＜0.05.

### 3.2 Genetic features of the ALS patients

One hundred and fifteen patients with ALS completed genetic testing, and 22 of them were detected to carry causative genetic variants, contributing to 19.13% (22/115) of all ALS patients. The most common mutant gene was *SOD1*, followed by *OPTN* and *CACNA1H*. Mutations that we identified are listed in Table S2.

### 3.3 Group comparison of ^18^F‐FDG‐PET data

#### 3.3.1 Patients with ALS versus HCs (height threshold at *p* < 0.001, *p* < 0.05 FWE‐corrected at cluster level)

**FIGURE 1:**
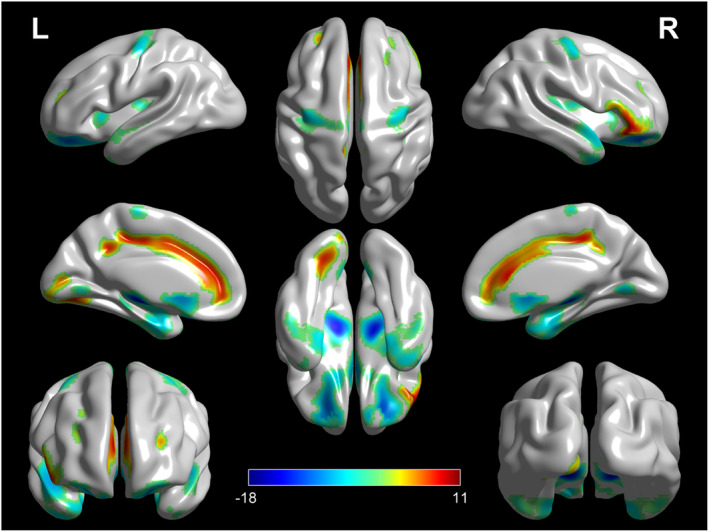
Patients with ALS versus HCs (*height threshold at p <* 0.001, *p <* 0.05 *FWE‐corrected at cluster level*). The regions showing statistically significant relative hypometabolism or hypermetabolism in patients with ALS as compared with HCs are reported on the brain surface.

Compared with HCs, patients with ALS showed relative hypometabolism in the bilateral temporal lobe, precentral gyrus, basal ganglia, midbrain, and cerebellum as compared with HCs (Figure 1; Table 2). Some regions with relatively increased metabolism in ALS were found in the left anterior cingulate, occipital lobe, and bilateral prefrontal lobe as compared with HCs (Figure 1; Table 2).

**TABLE 2:** Clusters showing a statistically significant relative hypermetabolism or hypometabolism in ALS patients as compared to HCs.

P (FWE‐corrected)	Cluster extent	T‐score	Peak coordinates (x, y, z) (mm)			Anatomical region	Cortical region	BA
0.000	6692	10.45	−14	44	0	Left limbic lobe	Anterior cingulate	
		10.09	−30	−36	36	Left frontal lobe	Subgyral	
		9.99	32	−32	36	Right frontal lobe	Subgyral	
0.002	722	10.21	48	32	−8	Right frontal lobe	Inferior frontal gyrus	
		9.17	52	34	0	Right frontal lobe	Inferior frontal gyrus	
		7.43	56	26	8	Right frontal lobe	Inferior frontal gyrus	
0.006	587	7.62	−24	−74	−6	Left occipital lobe	Subgyral	
		5.27	−10	−82	8	Left occipital lobe	Cuneus	17
		4.11	−10	−92	−4	Left occipital lobe	Lingual gyrus	
0.024	407	8.57	−50	30	−2	Left frontal lobe	Inferior frontal gyrus	
		7.25	−44	28	−10	Left frontal lobe	Inferior frontal gyrus	47
		7.03	−54	20	8	Left frontal lobe	Inferior frontal gyrus	45
0.000	26,535	−17.59	−42	−28	−28	Left temporal lobe	Subgyral	
		−17.25	−24	−2	12	Left sublobar	Lentiform nucleus	
		−16.66	42	−32	−28	Right temporal lobe	Fusiform gyrus	36
0.000	2012	−5.86	−36	−20	64	Left frontal lobe	Precentral gyrus	
		−5.67	−32	−14	52	Left frontal lobe	Precentral gyrus	
		−5.61	32	−14	52	Right frontal lobe	Precentral gyrus	

Abbreviations: ALS, amyotrophic lateral sclerosis; BA, Brodmann area; FWE, Family‐wise error; HCs, healthy controls.

#### 3.3.2 Patients with genetic ALS versus patients with nongenetic ALS (height threshold at *p* < 0.005, *p* < 0.05 FWE‐corrected at cluster level)

**FIGURE 2:**
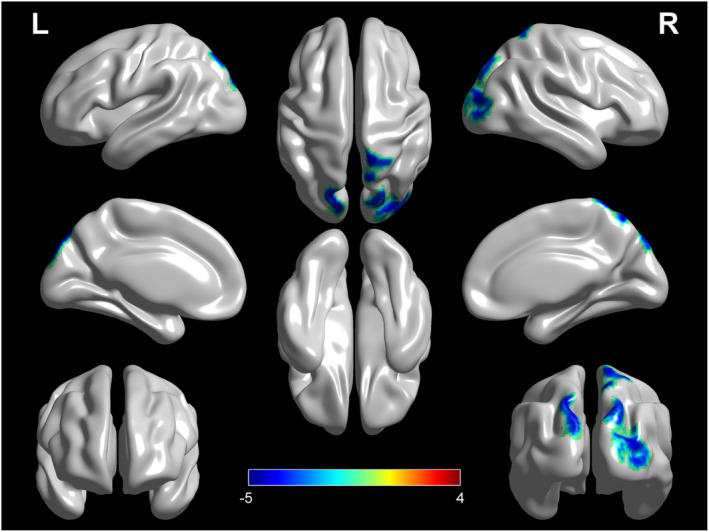
Patients with genetic ALS versus patients with nongenetic ALS (*height threshold* at *p* < 0.005, *p* < 0.05 *FWE‐corrected at cluster level*). The regions showing statistically significant relative hypometabolism in patients with genetic ALS as compared with patients with nongenetic ALS are reported on the brain surface. ALS, amyotrophic lateral sclerosis.

Since we did not identify any significant difference when setting the threshold at *p <* 0.001, we performed an exploratory analysis with the height threshold at *p* < 0.005. The genetic ALS group showed a cluster of relative hypometabolism, including the right precuneus, postcentral gyrus, and middle occipital gyrus as compared with the nongenetic ALS group (Figure 2; Table 3). No cluster of relative hypermetabolism was found in the genetic ALS group as compared to the nongenetic ALS group.

**TABLE 3:** Clusters showing a statistically significant relative hypometabolism in genetic ALS patients as compared to nongenetic ALS patients.

P (FWE‐corrected)	Cluster extent	T‐score	Peak coordinates (x, y, z) (mm)			Anatomical region	Cortical region	BA
0.000	2347	−4.57	8	−64	60	Right parietal lobe	Precuneus	7
		−4.23	14	−50	72	Right parietal lobe	Postcentral gyrus	7
		−4.13	32	−88	18	Right occipital lobe	Middle occipital gyrus	

Abbreviations: ALS, amyotrophic lateral sclerosis; BA, Brodmann area; FWE, Family‐wise error.

#### 3.3.3 Patients with genetic ALS versus HCs (height threshold at *p* < 0.001, *p* < 0.05 FWE‐corrected at cluster level)

**FIGURE 3:**
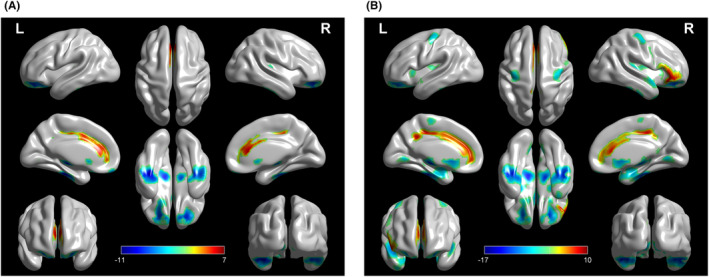
Patients with genetic ALS versus HCs and patients with nongenetic versus HCs (*height threshold at p <* 0.005, *p <* 0.05 *FWE‐corrected at cluster level*). (A) The regions showing statistically significant relative hypometabolism or hypermetabolism in patients with genetic ALS as compared with HCs are reported on the brain surface. (B) The regions showing statistically significant relative hypometabolism or hypermetabolism in patients with nongenetic ALS as compared with HCs are reported on the brain surface. ALS, amyotrophic lateral sclerosis; HCs, healthy controls.

Patients with genetic ALS showed relative hypometabolism in the bilateral sublobar, parahippocampal gyrus, occipital lobe, and cerebellum, and right temporal lobe as compared with HCs (Figure 3A; Table S3). A cluster of hypermetabolism was found in the bilateral frontal lobe and left sublobar in patients with genetic ALS as compared with HCs (Figure 3A; Table S3).

#### 3.3.4 Patients with nongenetic ALS versus HCs (height threshold at *p* < 0.001, *p* < 0.05 FWE‐corrected at cluster level)

Patients with nongenetic ALS showed relative hypometabolism in the left temporal lobe and precentral gyrus and bilateral sublobar (Figure 3B; Table S4). We identified a cluster of relative hypermetabolism in patients with nongenetic ALS as compared to HCs, including the left cingulate gyrus and occipital lobe and bilateral inferior frontal gyrus (Figure 3B; Table S4).

#### 3.3.5 ALS patients with sensory disturbance versus ALS patients with sensory normal (height threshold at *p* < 0.001, *p* < 0.05 FWE‐corrected at cluster level)

ALS patients with sensory disturbance showed hypometabolism in postcentral gyrus, precentral gyrus and other frontal–parietal lobe regions. (Figure S2; Table S7).

#### 3.3.6 ALS patients with sensory disturbance versus HCs (height threshold at *p* < 0.001, *p* < 0.05 FWE‐corrected at cluster level)

ALS patients with sensory abnormal showed hypometabolism in postcentral gyrus too. (Figure S3a; Table S8).

#### 3.3.7 ALS patients with sensory normal versus HCs (height threshold at *p* < 0.001, *p* < 0.05 FWE‐corrected at cluster level)

ALS patients showed relative hypometabolism in sublobar and temporal lobe and relative hypermetabolism in frontal lobe and limbic system as compared with HCs. There were no differences of metabolism between ALS patients with sensory normal and HCs in postcentral gyrus. (Figure S3b; Table S9).

## 4 DISCUSSION

In the present study, we specifically investigated brain metabolic disturbances in ALS patients and explored the relationship between brain metabolism and genotypes in a Chinese ALS cohort. As compared to HCs, we found hypometabolism in the primary motor cortex, frontal lobe, and temporal lobe in ALS patients in agreement with the results of previous studies.^9^, ^24^ Interestingly, as compared with HCs, we found that ALS patients also showed hypometabolism in the midbrain and cerebellum, which was inconsistent with previous studies.^9^, ^14^ Moreover, we also found that patients with genetic ALS showed hypometabolism in the right parietal and occipital lobe as compared to patients with nongenetic ALS.

Although previous studies have investigated the pattern of brain metabolism in ALS, an ^18^F‐FDG‐PET study in a large Chinese ALS cohort is still lacking. We performed a brain metabolic signature in ALS patients in the mainland of China in this study. And we found relative hypometabolism in the precentral gyrus of the frontal lobe and temporal lobe as described in previous studies.^9^, ^24^ Our findings further strengthen the evidence that ^18^F‐FDG‐PET could be used as a biomarker to evaluate the degeneration of upper motor neurons and cognitive dysfunction in ALS.

Interestingly, we found brain hypometabolism in the region of midbrain and cerebellum in patients with ALS as compared with HCs (Figure S1), which was inconsistent with previous ^18^F‐FDG‐PET studies.^9^, ^14^ Most of previous studies revealed the relative hypermetabolism in midbrain and cerebellum in ALS patients.^8^, ^9^, ^25^, ^26^ Some researchers suggest that hypermetabolism in the midbrain and cerebellum may be resulted by the astrocytosis and activated microglia.^9^, ^27^ Nevertheless, theoretically, ALS is a neurodegenerative disease characterized by the progressive loss of motor neurons in the brain, brainstem, and spinal cord,^1^, ^3^ thus the expected effect of ALS is hypometabolism in the midbrain caused by neuronal loss. Recently, an MRI study disclosed that patients with ALS exhibited focal cerebellar degeneration and cerebro‐cerebellar connectivity alterations.^28^ As with hypometabolism in the frontal and temporal in ALS,^10^, ^12^, ^27^ the degeneration of the cerebellum and midbrain inevitably leads to a reduction in tissue metabolic rate. Hence, we proposed a hypothesis that the metabolic states of the midbrain and cerebellum in patients with ALS are determined by which of the two pathological states of inflammation and degeneration is dominant. Further postmortem or specific PET tracer studies are required to validate the hypothesis.

In this study, 115 patients with ALS underwent genetic test. The most common mutant gene was *SOD1*, followed by *OPTN* and *CACNA1H*. This result was in line with previous studies in China.^16^, ^18^ As we all know, *SPG11* is a common AR‐inherited ALS causative gene.^29^ However, no mutations were detected in *SPG11*. There are two possible reasons listed below. First, in our cohort, none of the patients with ALS have autosomal recessive family history. Second, the AR‐inherited mutations in *SPG11* in Chinese ALS patients were rare.^16^, ^18^

As compared with HCs, the cluster of relative hypometabolism of patients with nongenetic ALS was major located in the temporal and frontal cortex, in line with the previous studies.^9^, ^24^ As compared with HCs, patients with genetic ALS showed relative hypometabolism in the occipital lobe, sublobar, and limbic lobe, which indicated that genetic ALS patients showed a specific brain metabolism signature.

Another interesting finding was hypermetabolism in the postcentral gyrus in patients with genetic ALS. The primary somatosensory cortex is located in the postcentral gyrus and widely interconnected with other brain regions, including the primary motor cortex. A previous study found that the number of neurons in the motor cortex and the somatosensory cortex were a positively correlated in ALS, suggesting that the somatosensory cortex is affected, once the degeneration of the motor cortex is initiated.^30^ These findings were also supported by other clinical researches on ALS. An MRI study found that patients with ALS showed parietal lobe atrophy during disease progression.^31^ Functional evaluation of the sensory cortex in patients with ALS using the high‐frequency somatosensory evoked potentials (HF‐SEP) disclosed significant somatosensory cortex dysfunction in patients with a disease duration of more than 2 years.^32^ In familial ALS patients, a more frequent occurrence of sensory features at presentation was reported.^33^ A previous study found that sensory disturbance was a more frequent feature in *C9orf72*‐associated ALS patients than non*C9orf72*‐associated ALS patients.^34^ Numerous studies reported that patients with ALS carrying *SOD1* causative mutations were more likely to have sensory abnormalities during the course of the disease.^35^, ^36^, ^37^ Consistent with previous studies, we also found the incidence of sensory disturbance in genetic ALS patients was higher than nongenetic ALS patients. Moreover, we further found that ALS patients with sensory disturbance displayed hypometabolism in postcentral gyrus and other regions of frontal–parietal lobe. ALS patients with sensory normal have no significant changes of metabolism in postcentral gyrus as compared with HCs. We provide direct and indirect evidence on the anatomo‐clinical correlations between hypometabolism in sensory brain regions and sensory disturbances. Recently, a neuroimaging study confirmed the degeneration of somatosensory, in ALS, which is more marked in *C9orf72* positive patients.^38^ A study about clusters of anatomical disease‐burden patterns in ALS confirmed the imaging signatures of sensory cortex could be distinct disease subtypes.^39^ Thus, these studies suggest that patients with ALS have somatosensory cortex involvement, although the molecular mechanism is unclear. Combining with our results, we hypothesize that genetic factors may be an underlying cause of sensory disturbances in ALS.

Similar to the previous PET studies,^9^, ^40^ we also found hypometabolism in occipital lobes in genetic ALS patients as compared with nongenetic ALS patients. Several MRI studies reported reductions of cortical thickness, gray matter volume, and functional connectivity in occipital lobes in patients with ALS.^41^, ^42^, ^43^ The above studies indicated the abnormal alterations in the occipital lobes of ALS patients, while the molecular mechanism is unknown. *C9orf72*‐linked FTD‐ALS patients were found to present parietal and occipital lobe atrophy using structural MRI scans.^44^ A postmortem study found that two aberrant *SOD1* mRNAs were detected from occipital cortex of ALS patients.^45^ These findings suggested that mutations in known causative ALS genes may affect the metabolism of the occipital lobes of ALS patients. Neuroaxonal retinal abnormalities were detected in neurodegenerative diseases like Parkinson's Disease, progressive supranuclear palsy, multiple system atrophy by optical coherence tomography.^46^, ^47^ Moreover, a previous study reported that retinal nerve fiber layer thinning was associated with the atrophy of occipital lobe in Alzheimer's disease.^48^ These studies indicate that pathological changes of visual pathway were solid evidence in neurodegenerative diseases. Recently, reported alteration of the retinal nerve in ALS was reported implied that the ALS visual pathway may be damaged.^49^ The effects of genetic factors on pathological changes in the visual pathway in ALS require more investigation.

There are several limitations in our study. First, the relatively small sample size of the genetic group might influence the results of our study. Second, ALS patients were recruited from a single center in China and our findings regarding the correlations between genotype and brain metabolism in Chinese ALS patients cannot be generalized to European or American ALS populations. Third, we could not correct the effects of cortical atrophy, since not all patients underwent brain MRI scans in this study. Nevertheless, previous studies showed that the result of brain metabolism is relatively independent of cortical atrophy.^50^ Fourth, since the specific functional scale of the occipital lobe in our study is lacking, we were unable to assess the difference in the function of the occipital lobe between patients with genetic ALS and nongenetic ALS. The correlation between clinical phenotype and hypometabolism in the occipital lobe in ALS needs further studies to discover. Moreover, cognitive screening is not available in part of ALS patients in our study. It does make some bias of the results in our study. Some patients overlapping ALS and other dementia diseases were not excluded in this study due to the lack of cognitive screening.

In conclusion, our observations strengthen the evidence that ^18^F‐FDG‐PET is a reliable tool to assess the motor in ALS, and our investigation provided unprecedented evidence of relative hypometabolism in midbrain and cerebellum in ALS patients as compared with HCs. Genetic ALS patients showed a specific signature of brain metabolism and a higher incidence of sensory disturbance, indicating that genetic factors may be an underlying cause affecting the brain metabolism and increasing the risk of sensory disturbance in ALS. Further studies may be required to confirm our preliminary findings.

## CONFLICT OF INTEREST STATEMENT

The authors declare that there is no conflict of interest associated with the contents of this article.

## Supporting information


Appendix S1

## Data Availability

Datasets analyzed in this study are not publicly available. Further information about the datasets is available from the senior author (J.W.) and (S.H.) on reasonable request.
