# Supplementary material for: Brain metabolic signatures in patients with genetic and nongenetic amyotrophic lateral sclerosis
Source: CNS Neurosci Ther. 2023 Mar 27;29(9):2530–9. doi: 10.1111/cns.14193 (PMC10401109; doi:10.1111/cns.14193)
Supplement: Supplementary file 1 — Appendix S1 [file CNS-29-2530-s001.docx]

**Supplementary File**

**Article title:** Brain metabolic signatures in patients with genetic and non-genetic amyotrophic lateral sclerosis

**Journal name:** *CNS Neuroscience & Therapeutics*

- **Table S1** The list of ALS genes analyzed in this study.
- **Table S2** Clinical features of patients carrying ALS gene mutations.
- **Table S3** Clusters showing a statistically significant relative hypermetabolism or hypometabolism in genetic ALS patients as compared to HCs.
- **Table S4** Clusters showing a statistically significant relative hypermetabolism or hypometabolism in non-genetic ALS patients as compared to HCs.
- **Table S5** The difference of demographic and clinical features between sensory abnormal ALS and sensory normal ALS.
- **Table S6** The difference of demographic and clinical features between sensory abnormal ALS and HCs and between sensory normal ALS and HCs.
- **Table S7** Clusters showing a statistically significant relative hypometabolism in patients with ALS with sensory disturbance as compared to patients with ALS with sensory normal.
- **Table S8** Clusters showing a statistically significant relative hypermetabolism or hypometabolism in patients with ALS with sensory disturbance as compared to HCs.
- **Table S9** Clusters showing a statistically significant relative hypermetabolism or hypometabolism in patients with ALS with sensory normal as compared to HCs.
- **FIGURE S1.** Patients with ALS versus HCs *(height threshold at p < 0.001, p < 0.05 FWE-corrected at cluster level)*.
- **FIGURE S2.** Patients with ALS with sensory disturbance versus patients with ALS with sensory normal (height threshold at p < 0.005, p < 0.05 FWE-corrected at cluster level).
- **FIGURE S3.** Patients with ALS with sensory disturbance versus HCs and patients with ALS with sensory normal versus HCS *(height threshold at p < 0.005, p < 0.05 FWE-corrected at cluster level)*.

**Table S1** The list of ALS genes analyzed in this study

| Gene list | | | | | | |
| --- | --- | --- | --- | --- | --- | --- |
| *SOD1* | *TARDBP* | *C9orf72* | *ATXN2* | *FUS* | *ALS2* | *OPTN* |
| *SETX* | *FIG4* | *MATR3* | *DAO* | *SQSTM1* | *DCTN1* | *ERLIN1* |
| *ANXA11* | *C21orf2* | *VRK1* | *TAF15* | *DNAJC7* | *NEFH* | *hnRNPA2B1* |
| *VAPB* | *VCP* | *ERBB4* | *NEK1* | *GLT8D1* | *GRN* | *ATP13A2* |
| *ANG* | *CHMP2B* | *TUBA4A* | *GLE1* | *TBK1* | *GARS* | *CACNA1H* |
| *UBQLN2* | *ELP3* | *hnRNPA1* | *KIF5A* | *CHCHD10* | *TIA1* | *DJ1* |
| *EWSR1* | *PRPH* | *SIGMAR1* | *C19orf12* | *SPG11* | *SYNE1* | *CCNF* |
| *SS18L1* | *PFN1* | *NOTCH3* |  |  |  |  |

**Table S2** Clinical features of patients carrying ALS gene mutations

| No | Sex | Age | Site of onset | Clinical phenotype | Mutant gene | Nucleotide change | Amino acid  change | ReVe | Sensory disturbances |
| --- | --- | --- | --- | --- | --- | --- | --- | --- | --- |
| 1 | Male | 40 | Bulbar | LMN+UMN | *VCP* | c.555A>C | p.E185D | 0.77 | Yes |
| 2 | Male | 54 | Upper limb | LMN | *OPTN* | c.1690G>C | p.D564H | 0.94 | No |
| 3 | Male | 56 | Upper limb | LMN+UMN | *ERLIN1* | c.C671A | p.A224E | 0.86 | Yes |
| 4 | Male | 54 | Lower limb | LMN+UMN | *CACNA1H* | c.2849C>T | p.T950I | 0.83 | No |
| 5 | Male | 71 | Bulbar | Bulbar paralysis | *OPTN* | c.481G>A | p.V161M | 0.75 | No |
| 6 | Male | 51 | Lower limb | LMN | *SOD1* | c.341T>C | p.I114T | 0.99 | Yes |
| 7 | Female | 38 | Bulbar | LMN+UMN | *NOTCH3* | c.515G>A | p.G172D | 0.87 | No |
| 8 | Male | 51 | Lower limb | LMN+UMN | *MATR3* | c.1991A>C | p.E664A | 0.72 | Yes |
| 9 | Male | 53 | Upper limb | LMN+UMN | *NEK1*  *TBK1* | c.1693_1694del TTC  c.1834delT | p.E565fs  p.F612fs | -  - | No |
| 10 | Male | 67 | Upper limb | LMN | *NOTCH3* | c.625G>A | p.G209R | 0.94 | No |
| 11 | Male | 65 | Lower limb | LMN | *SOD1* | c.335G>A | p.C112Y | 0.85 | No |
| 12 | Male | 49 | Lower limb | LMN+UMN | *NEK1* | c.437A>C | p.D146A | 0.99 | No |
| 13 | Female | 37 | Lower limb | LMN+UMN | *SOD1* | c.113G>T | p.G38V | 0.99 | No |
| 14 | Male | 50 | Lower limb | LMN+UMN | *NEFH* | c.674A>T | p.E225V | 0.79 | Yes |
| 15 | Male | 55 | Lower limb | LMN+UMN | *FIG4* | c.1199A>G | p.Y400C | 0.80 | No |
| 16 | Male | 50 | Upper limb | LMN+UMN | *ERBB4* | c.158A>G | p.Y53C | 0.99 | No |
| 17 | Male | 27 | Upper limb | LMN+UMN | *SOD1* | c.443G>A | p.G148D | 1.00 | No |
| 18 | Male | 62 | Upper limb | LMN | *GARS* | c.2T>A | p.M1K | 0.83 | No |
| 19 | Male | 61 | Upper limb | LMN+UMN | *TP73* | c.946T>C | p.F316L | 0.98 | No |
| 20 | Female | 63 | Lower limb | LMN+UMN | *SOD1* | c. 14C>T | p.A5V | 0.93 | No |
| 21 | Female | 56 | Bulbar | Bulbar paralysis | *CACNA1H* | c.4684C>T | p.R1562W | 0.79 | No |
| 22 | Male | 38 | Lower limb | LMN | *OPTN* | c.1481T>G | p.L494W | 0.74 | No |

**Abbreviations:** LMN, lower motor neuron; UMN, upper motor neuron.

**Table S3** Clusters showing a statistically significant relative hypermetabolism or hypometabolism in genetic ALS patients as compared to HCs.

| P (FWE-corrected) | Cluster extent | T-score | Peak coordinates  (x, y, z) (mm) | | | Anatomical region | Cortical region | BA |
| --- | --- | --- | --- | --- | --- | --- | --- | --- |
| 0.000 | 3717 | 6.72 | -30 | -36 | 36 | Left frontal lobe | Sub-Gyral |  |
|  |  | 6.68 | 34 | -30 | 36 | Right frontal lobe | Sub-Gyral |  |
|  |  | 6.62 | -2 | 26 | 14 | Left sub-lobar | Extra-nuclear |  |
| 0.000 | 9063 | -10.54 | -12 | -18 | 0 | Left sub-lobar | Thalamus |  |
|  |  | -10.09 | -24 | -4 | 10 | Left sub-lobar | Lentiform nucleus |  |
|  |  | -9.68 | 14 | -16 | 0 | Right sub-lobar | Thalamus |  |
| 0.000 | 1699 | -6.43 | -14 | -50 | -36 | Left cerebellum | anterior lobe |  |
|  |  | -5.82 | -4 | -52 | -32 | Left cerebellum | anterior lobe |  |
|  |  | -5.79 | 4 | -50 | -34 | Right cerebellum | Fourth ventricle |  |
| 0.000 | 1034 | -6.28 | 2 | -96 | 4 | Right occipital lobe | Cuneus | 18 |
|  |  | -4.89 | 2 | -80 | -14 | Right occipital lobe | - |  |
|  |  | -4.64 | -2 | -68 | -4 | Left occipital lobe | - |  |
| 0.004 | 633 | -10.43 | -40 | -28 | -28 | Left limbic lobe | Parahippocampa gyrus |  |
| 0.006 | 563 | -9.09 | 40 | -30 | -28 | Right limbic lobe | Parahippocampa gyrus | 36 |
|  |  | -8.08 | 32 | -22 | -30 | Right limbic lobe | Parahippocampa gyrus | 36 |
|  |  | 5.36 | 52 | -22 | -32 | Right temporal lobe | Fusiform gyrus | 20 |

**Abbreviations:** ALS, amyotrophic lateral sclerosis; BA, Broadmann area; FWE, Family-wise error; HCs, healthy controls.

**Table S4** Clusters showing a statistically significant relative hypermetabolism or hypometabolism in non-genetic ALS patients as compared to HCs.

| P (FWE-corrected) | Cluster extent | T-score | Peak coordinates  (x, y, z) (mm) | | | Anatomical region | Cortical region | BA |
| --- | --- | --- | --- | --- | --- | --- | --- | --- |
| 0.000 | 6035 | 10.03 | -14 | -44 | 34 | Left limbic lobe | Cingulate gyrus |  |
|  |  | 9.79 | -14 | 44 | 0 | Left limbic lobe | Anterior cingulate |  |
|  |  | 9.28 | -30 | -36 | 36 | Left frontal lobe | Sub-Gyral |  |
| 0.002 | 737 | 10.06 | 48 | 32 | -8 | Right frontal lobe | Inferior frontal gyrus |  |
|  |  | 9.10 | 52 | 34 | 0 | Right frontal lobe | Inferior frontal gyrus |  |
|  |  | 7.35 | 56 | 26 | 8 | Right frontal lobe | Inferior frontal gyrus | 45 |
| 0.009 | 513 | 7.08 | -24 | -74 | -6 | Left occipital lobe | Sub-Gyral |  |
|  |  | 5.20 | -10 | -82 | 8 | Left occipital lobe | Cuneus | 17 |
|  |  | 3.98 | -10 | -92 | -6 | Left occipital lobe | Lingual gyrus |  |
| 0.021 | 415 | 8.47 | -50 | 30 | -2 | Left frontal lobe | Inferior frontal gyrus |  |
|  |  | 7.42 | -44 | 30 | -10 | Left frontal lobe | Inferior frontal gyrus |  |
|  |  | 6.94 | -54 | 22 | 10 | Left frontal lobe | Inferior frontal gyrus |  |
| 0.000 | 26981 | -16.73 | -42 | -28 | -28 | Left temporal lobe | Sub-Gyral |  |
|  |  | -16.71 | -24 | -4 | 12 | Left sub-lobar | Lentiform nucleus |  |
|  |  | -16.16 | 26 | -2 | 12 | Right sub-lobar | Lentiform nucleus |  |
| 0.000 | 2074 | -5.65 | -12 | -20 | 64 | Left frontal lobe | Sub-Gyral |  |
|  |  | -5.57 | -32 | -14 | 52 | Left frontal lobe | Precentral gyrus |  |
|  |  | -5.54 | -36 | -20 | 64 | Left frontal lobe | Precentral gyrus |  |

**Abbreviations:** ALS, amyotrophic lateral sclerosis; BA, Broadmann area; FWE, Family-wise error; HCs, healthy controls.

**Table S5** The difference of demographic and clinical features between sensory abnormal ALS and sensory normal ALS.

|  | Sensory abnormal ALS | Sensory normal ALS | *P* |
| --- | --- | --- | --- |
| Number | 12 | 134 |  |
| Age at PET (years) | 51.41 ± 6.75 | 55.83 ± 11.03 | 0.081 **^a^** |
| Sex |  |  | 0.196 **^b^** |
| Male (%) | 10 (83.3) | 87 (64.9) |  |
| Female (%) | 2 (16.7) | 47 (35.1) |  |
| Years of education | 8.01 ± 3.11 | 8.87 ± 3.71 | 0.635 **^a^** |
| Age at onset (years) | 50.75 ± 6.58 | 55.10 ± 10.42 | 0.168 **^b^** |
| Diagnostic category (Revised El Escorial criteria 2015) |  |  | 0.151**^c^** |
| Definite ALS (%) | 1 (8.3) | 31 (23.1) |  |
| Probable ALS (%) | 5 (41.7) | 26 (19.4) |  |
| Laboratory support probable ALS (%) | 6 (50.0) | 77 (57.5) |  |
| Site of onset |  |  | 0.884**^c^** |
| Bulbar onset (%) | 3 (25.0) | 31 (23.1) |  |
| Spinal onset (%) | 9 (75.0) | 103 (76.9) |  |
| Disease duration (months) | 10.17 ± 5.97 | 14.82 ± 15.63 | 0.308 **^b^** |
| ALSFRS-R | 38.33 ± 6.10 | 38.90 ± 6.67 | 0.776 **^b^** |
| Genetic status |  |  | **0.007 ^c^** |
| Positive | 5 (41.7) | 17 (12.7) |  |
| Negative | 7 (58.3) | 117 (87.3) |  |

**Abbreviations:** ALS, Amyotrophic lateral sclerosis; ALSFRS-R, Amyotrophic Lateral Sclerosis Functional Rating Scale–Revised; HCs, healthy controls.

**^a^** Student’s t-test, *P*＜0.05 was considered significant; **^b^** Mann-Whitney U test, *P*＜0.05 was considered significant; **^c^**χ2 test, *P*＜0.05 was considered significant

**Table S6** The difference of demographic and clinical features between sensory abnormal ALS and HCs and between sensory normal ALS and HCs

|  | Sensory abnormal ALS patients | HCs | *P* | Sensory normal ALS | HCs | *P* |
| --- | --- | --- | --- | --- | --- | --- |
| Number | 12 | 128 |  | 134 | 128 |  |
| Age at PET (years) | 51.41 ± 6.75 | 55.24 ± 7.75 | 0.101 **^a^** | 55.83 ± 11.03 | 55.24 ± 7.75 | 0.081 **^a^** |
| Sex |  |  | 0.292 |  |  | 0.196 **^c^** |
| Male (%) | 10 (83.3) | 88 (68.8) |  | 87 (64.9) | 88 (68.8) |  |
| Female (%) | 2 (16.7) | 40 (31.2) |  | 47 (35.1) | 40 (31.2) |  |
| Years of education | 8.01 ± 3.11 | 8.82 ± 2.77 | 0.361 | 8.87 ± 3.71 | 8.82 ± 2.77 | 0.635 **^b^** |

**Abbreviations:** ALS, Amyotrophic lateral sclerosis; ALSFRS-R, Amyotrophic Lateral Sclerosis Functional Rating Scale–Revised; HCs, healthy controls.

**^a^** Student’s t-test, *P*＜0.05 was considered significant; **^b^** Mann-Whitney U test, *P*＜0.05 was considered significant; **^c^**χ2 test, *P*＜0.05 was considered significant

**Table S7** Clusters showing a statistically significant relative hypometabolism in patients with ALS with sensory disturbance as compared to patients with ALS with sensory normal.

| P (FWE-corrected) | Cluster extent | T-score | Peak coordinates  (x, y, z) (mm) | | | Anatomical region | Cortical region | BA |
| --- | --- | --- | --- | --- | --- | --- | --- | --- |
| 0.000 | 1209 | -5.17 | 52 | -26 | 32 | Right parietal lobe | Postcentral gyrus | 2 |
|  |  | -4.49 | 32 | 8 | 22 | Right frontal lobe | Sub-Gyral |  |
|  |  | -4.01 | 38 | -16 | 28 | Right frontal lobe | Precentral gyrus |  |
| 0.002 | 710 | -4.62 | 20 | -44 | 46 | Right parietal lobe | Sub-Gyral |  |
|  |  | -4.45 | 34 | -42 | 52 | Right parietal lobe | Sub-Gyral |  |
|  |  | -3.88 | 20 | -30 | 52 | Right frontal lobe | Sub-Gyral |  |

**Abbreviations:** ALS, amyotrophic lateral sclerosis; BA, Broadmann area; FWE, Family-wise error

**Table S8** Clusters showing a statistically significant relative hypermetabolism or hypometabolism in patients with ALS with sensory disturbance as compared to HCs.

| P (FWE-corrected) | Cluster extent | T-score | Peak coordinates  (x, y, z) (mm) | | | Anatomical region | Cortical region | BA |
| --- | --- | --- | --- | --- | --- | --- | --- | --- |
| 0.000 | 19893 | -11.50 | -14 | -20 | 0 | Left sub-lobar | Thalamus | 2 |
|  |  | -10.88 | 14 | -18 | -2 | Right brainstem | Midbrain |  |
|  |  | -10.15 | -24 | -6 | 12 | Left sub-lobar | Lentiform nucleus |  |
| 0.000 | 877 | -10.32 | -44 | -30 | -26 | Left temporal lobe | Fusiform gyrus | 20 |
|  |  | -4.38 | -28 | -38 | -42 | Left cerebellum | Cerebellar tonsil |  |
|  | 879 | -5.49 | -6 | -64 | 0 | Left occipital lobe | Lingual gyrus | 18 |
|  |  | -4.87 | 8 | -62 | -2 | Right occipital lobe | Lingual gyrus | 19 |
|  |  | -4.66 | 6 | -52 | 6 | Right limbic lobe | Posterior cingulate | 29 |
| 0.003 | 636 | 5.80 | -12 | 44 | -2 | Left limbic lobe | Anterior cingulate |  |
|  |  | 4.64 | 12 | 44 | 2 | Right frontal lobe | Sub-Gyral |  |
|  |  | 4.30 | 0 | 26 | 14 | Inter-Hemispheric | - |  |
| 0.025 | 378 | 5.58 | -50 | 30 | -2 | Left frontal lobe | Inferior frontal gyrus |  |
|  |  | 5.08 | -50 | 36 | 8 | Left frontal lobe | Inferior frontal gyrus |  |
|  |  | 4.96 | -54 | 20 | 8 | Left frontal lobe | Inferior frontal gyrus | 45 |

**Abbreviations:** ALS, amyotrophic lateral sclerosis; BA, Broadmann area; FWE, Family-wise error; HCs, healthy controls.

**Table S9** Clusters showing a statistically significant relative hypermetabolism or hypometabolism in patients with ALS with sensory normal as compared to HCs.

| P (FWE-corrected) | Cluster extent | T-score | Peak coordinates  (x, y, z) (mm) | | | Anatomical region | Cortical region | BA |
| --- | --- | --- | --- | --- | --- | --- | --- | --- |
| 0.000 | 19877 | -12.23 | -24 | -4 | 12 | Left Sub-lobar | Lentiform nucleus |  |
|  |  | -11.97 | 24 | -2 | 10 | Right Sub-lobar | Lentiform nucleus |  |
|  |  | -11.65 | 44 | -28 | -28 | Right temporal lobe | Fusiform gyrus |  |
| 0.000 | 1130 | -6.07 | -2 | -70 | -6 | Left cerebellum | - |  |
| 0.000 | 3609 | 8.68 | -30 | -36 | 36 | Left frontal lobe | Sub-Gyral |  |
|  |  | 8.07 | -14 | -44 | 34 | Left limbic lobe | Cingulate gyurs |  |
|  |  | 8.01 | -14 | 44 | 2 | Left frontal lobe | Sub-Gyral |  |
|  | 971 | 7.78 | 18 | -32 | 42 | Right limbic lobe | Cingulate gyurs |  |
|  |  | 6.94 | 28 | -40 | 36 | Right frontal lobe | Sub-Gyral |  |
|  |  | 6.90 | 32 | -30 | 38 | Right frontal lobe | Sub-Gyral |  |
|  | 374 | 7.60 | 46 | 30 | -10 | Right frontal lobe | Inferior frontal gyrus |  |
|  |  | 7.22 | 48 | 40 | -8 | Right frontal lobe | Middle frontal gyrus |  |
|  |  | 6.52 | 52 | 32 | 0 | Right frontal lobe | Inferior frontal gyrus |  |

**Abbreviations:** ALS, amyotrophic lateral sclerosis; BA, Broadmann area; FWE, Family-wise error; HCs, healthy controls.


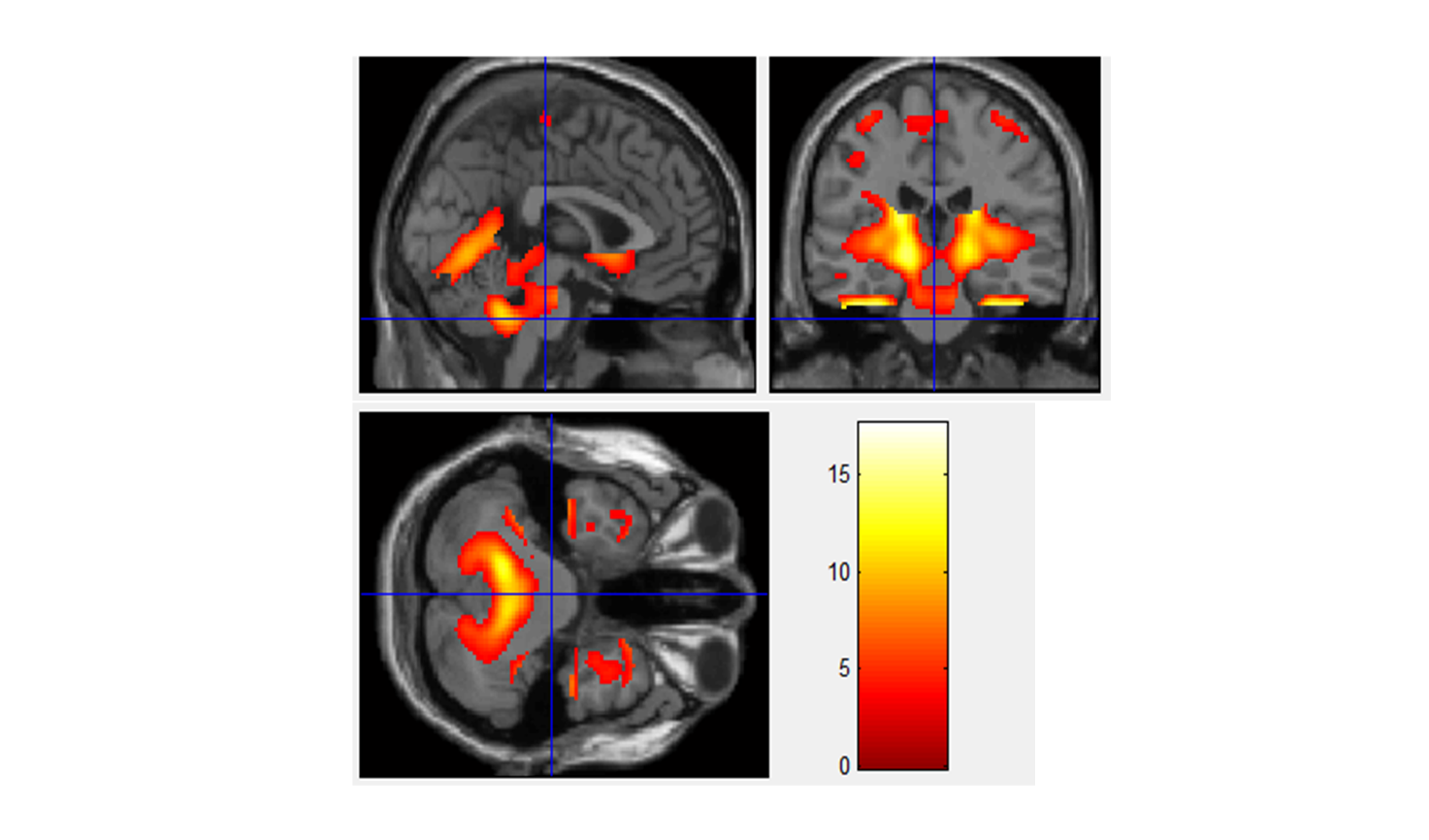


**FIGURE S1.** Patients with ALS versus HCs *(height threshold at p < 0.001, p < 0.05 FWE-corrected at cluster level)*. The regions showing statistically significant relative hypometabolism in patients with ALS as compared with HCs are reported on a brain magnetic resonance imaging template. ALS, amyotrophic lateral sclerosis; HCs, healthy controls.


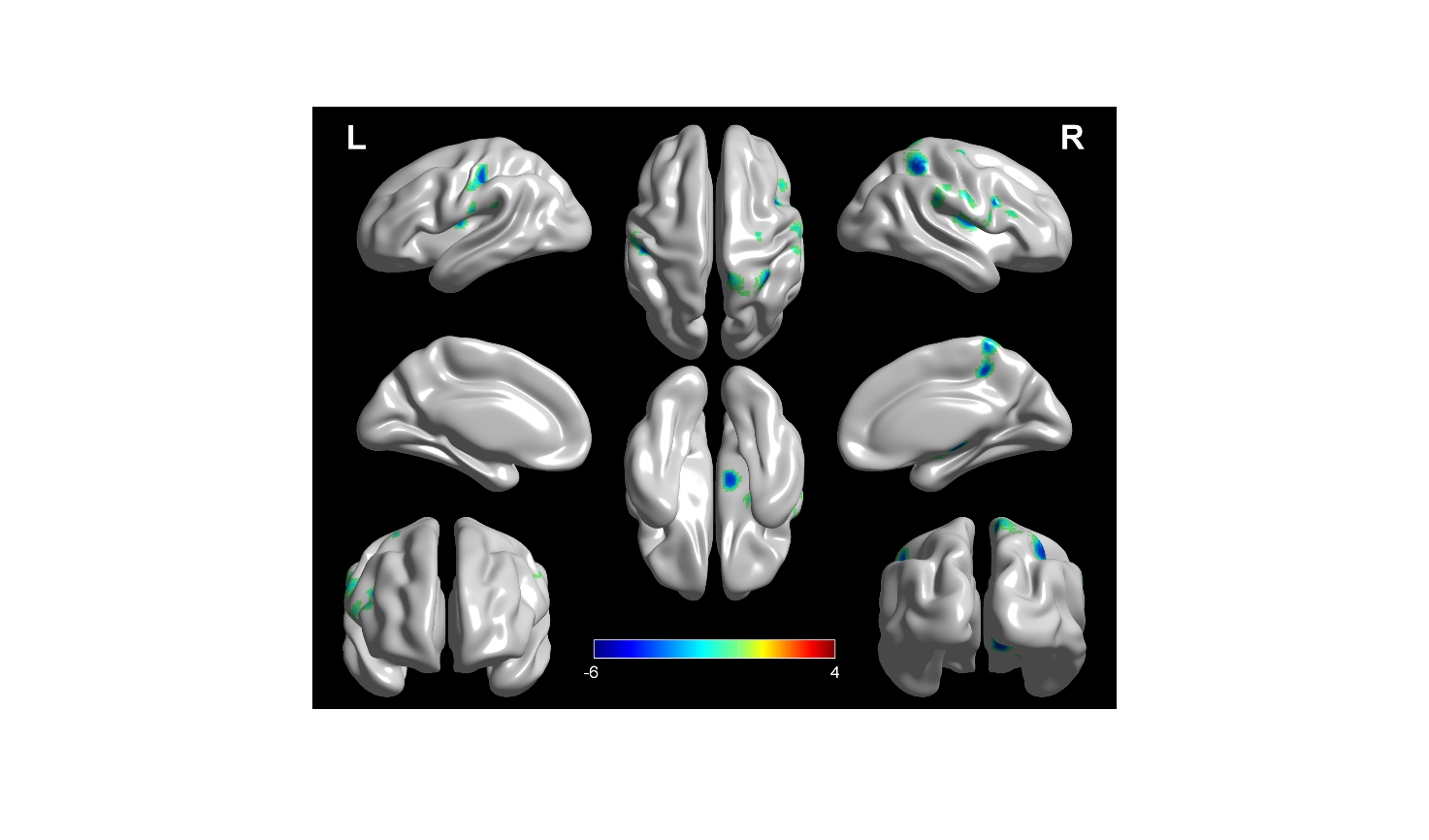


**FIGURE S2.** Patients with ALS with sensory disturbance versus patients with ALS with sensory normal *(height threshold at p < 0.005, p < 0.05 FWE-corrected at cluster level)*. The regions showing statistically significant relative hypometabolism in patients with ALS with sensory disturbance as compared with patients with ALS with sensory normal are reported on the brain surface.


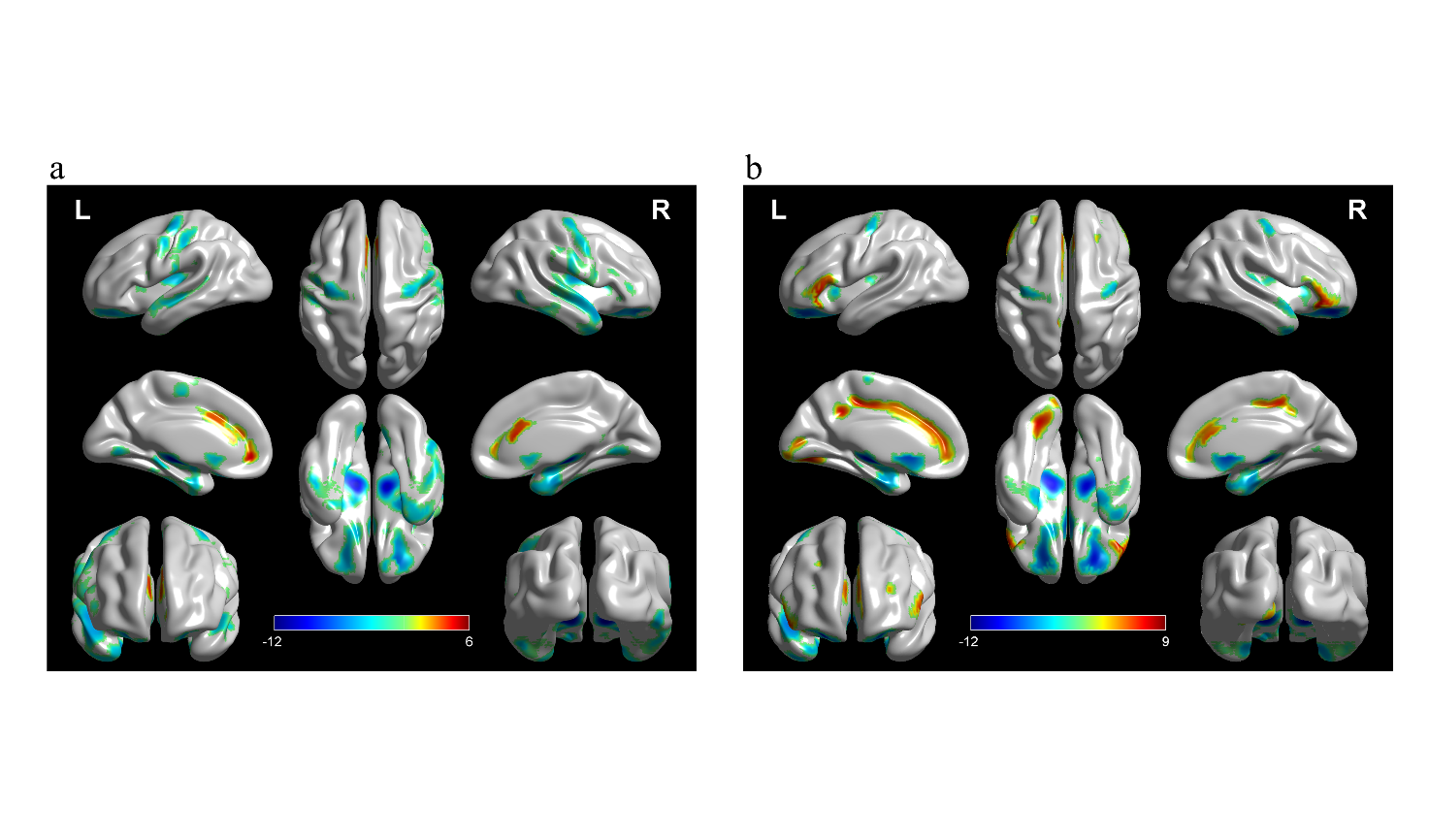


**FIGURE S3.** Patients with ALS with sensory disturbance versus HCs and patients with ALS with sensory normal versus HCS *(height threshold at p < 0.005, p < 0.05 FWE-corrected at cluster level)*. (a) The regions showing statistically significant relative hypometabolism in patients with ALS with sensory disturbance as compared HCs are reported on the brain surface. (b) The regions showing statistically significant relative hypometabolism in patients with ALS with sensory normal as compared with HCs are reported on the brain surface. ALS, amyotrophic lateral sclerosis; HCs, healthy controls.
